# Influence of coronary artery disease and subclinical atherosclerosis related polymorphisms on the risk of atherosclerosis in rheumatoid arthritis

**DOI:** 10.1038/srep40303

**Published:** 2017-01-06

**Authors:** Raquel López-Mejías, Alfonso Corrales, Esther Vicente, Montserrat Robustillo-Villarino, Carlos González-Juanatey, Javier Llorca, Fernanda Genre, Sara Remuzgo-Martínez, Trinidad Dierssen-Sotos, José A. Miranda-Filloy, Marco A. Ramírez Huaranga, Trinitario Pina, Ricardo Blanco, Juan J. Alegre-Sancho, Enrique Raya, Verónica Mijares, Begoña Ubilla, Iván Ferraz-Amaro, Carmen Gómez-Vaquero, Alejandro Balsa, Francisco J. López-Longo, Patricia Carreira, Isidoro González-Álvaro, J. Gonzalo Ocejo-Vinyals, Luis Rodríguez-Rodríguez, Benjamín Fernández-Gutiérrez, Santos Castañeda, Javier Martín, Miguel A. González-Gay

**Affiliations:** 1 Epidemiology, Genetics and Atherosclerosis Research Group on Systemic Inflammatory Diseases, Rheumatology Department, IDIVAL, Santander, Spain; 2 Rheumatology Department, Hospital Universitario la Princesa, IIS-IP, Madrid, Spain; 3 Rheumatology Department, Hospital Universitario Doctor Peset, Valencia, Spain; 4 Cardiology Department, Hospital Lucus Augusti, Lugo, Spain; 5 Department of Epidemiology and Computational Biology, School of Medicine, University of Cantabria, and CIBER Epidemiología y Salud Pública (CIBERESP), IDIVAL, Santander, Spain; 6 Division of Rheumatology, Hospital Universitario Lucus Augusti, Lugo, Spain; 7 Rheumatology Department, Hospital General Universitario de Ciudad Real, Ciudad Real, Spain; 8 Rheumatology Department, Hospital Clínico San Cecilio, Granada, Spain; 9 Rheumatology Division, Hospital Universitario de Canarias, Santa Cruz de Tenerife, Spain; 10 Department of Rheumatology, Hospital Universitario Bellvitge, Barcelona, Spain; 11 Department of Rheumatology, Hospital Universitario La Paz, Madrid, Spain; 12 Department of Rheumatology, Hospital General Universitario Gregorio Marañón, Madrid, Spain; 13 Department of Rheumatology, Hospital Universitario 12 de Octubre, Madrid, Spain; 14 Immunology Department, Hospital Universitario Marqués de Valdecilla, Santander, Spain; 15 Department of Rheumatology, Hospital Clínico San Carlos, Madrid, Spain; 16 Instituto de Parasitología y Biomedicina ‘López-Neyra’, CSIC, PTS Granada, Granada, Spain; 17 School of Medicine, University of Cantabria, Santander, Spain; 18 Cardiovascular Pathophysiology and Genomics Research Unit, School of Physiology, Faculty of Health Sciences, University of the Witwatersrand, Johannesburg, South Africa

## Abstract

A genetic component influences the development of atherosclerosis in the general population and also in rheumatoid arthritis (RA). However, genetic polymorphisms associated with atherosclerosis in the general population are not always involved in the development of cardiovascular disease (CVD) in RA. Accordingly, a study in North-American RA patients did not show the association reported in the general population of coronary artery disease with a series of relevant polymorphisms (*TCF21, LPA, HHIPL1, RASD1-PEMT, MRPS6, CYP17A1-CNNM2-NT5C2, SMG6-SRR, PHACTR1, WDR12* and *COL4A1-COL4A2)*. In the present study, we assessed the potential association of these polymorphisms with CVD in Southern European RA patients. We also assessed if polymorphisms implicated in the increased risk of subclinical atherosclerosis in non-rheumatic Caucasians (*ZHX2, PINX1, SLC17A4, LRIG1* and *LDLR)* may influence the risk for CVD in RA. 2,609 Spanish patients were genotyped by TaqMan assays. Subclinical atherosclerosis was determined in 1,258 of them by carotid ultrasonography (assessment of carotid intima media thickness and presence/absence of carotid plaques). No statistically significant differences were found when each polymorphism was assessed according to the presence/absence of cardiovascular events and subclinical atherosclerosis, after adjustment for potential confounder factors. Our results do not show an association between these 15 polymorphisms and atherosclerosis in RA.

A genetic component influences the development of atherosclerosis in the general population and also in patients with rheumatoid arthritis (RA)^1^,^2^. Several pieces of evidence support the hypothesis that both pathologies share many similarities and exhibit analogous pathophysiological mechanisms^3^. However, genetic polymorphisms associated with atherosclerosis in the general population are not always involved in the development of cardiovascular disease (CVD) in RA^2^. In this respect, a recent study performed in patients with RA from North-America did not disclose that a series of gene polymorphisms related to coronary artery disease in the general population^4^ were involved in the development of atherosclerotic disease in RA^5^. This study included, among others, the following polymorphisms: *TCF21* [*transcription factor 21*] rs12190287, *LPA [lipoprotein, Lp[a]]* rs3798220, *HHIPL1* [*Hedgehog interacting protein-like protein 1*] rs2895811, *RASD1 [RAS dexamethasone-induced 1]-PEMT* [*phosphatidylethanolamine N-methyltransferase*] rs12936587, *MRPS6 [mitocondrial ribosomal protein S6]* rs9982601, *CYP17A1 [Cytochrome P450, Family 17, Subfamily A, Polypeptide 1]-CNNM2 [Cyclin and CBS domain divalent metal cation transport mediator 2]-NT5C2* [*5*′*-nucleotidase, cytosolic II*] rs12413409, *SMG6 [SMG6 nonsense mediated mRNA decay factor]-SRR* [*serine racemase*] rs216172, *PHACTR1 [phosphatase and actin regulator 1]* rs12526453, *WDR12 [WD repeat domain 12]* rs6725887 and *COL4A1-COL4A2 [collagen type IV alpha 1- collagen type IV alpha 2]* rs4773144^5^.

In the present study, we assessed the potential association of these polymorphisms with CVD in Southern European individuals with RA. Moreover, we also assessed if other gene polymorphisms implicated in the increased risk of subclinical atherosclerosis in non-rheumatic Caucasian individuals^6^ may influence the risk for CVD in RA. These polymorphisms associated with subclinical atherosclerosis in the general population were the following: *ZHX2 [zinc fingers and homeoboxes 2]* rs11781551, *PINX1 [Pin2-interacting protein]* rs6601530 and *SLC17A4 [solute carrier family 17, member 4]* rs4712972 as significant signals associated with carotid intima-media thickness (cIMT) and *LRIG1* [*leucine-rich repeats and immunoglobulin-like domains 1*] rs17045031 and *LDLR [low density lipoprotein receptor]* rs6511720 as relevant polymorphisms involved in the presence of carotid plaques^6^.

## Patients and Methods

### Patients and Study Protocol

A set of 2,609 unrelated Spanish RA patients fulfilling the 2010 American College of Rheumatology classification criteria for RA^7^ was included in our study. Blood samples were obtained from patients recruited from Hospital Lucus Augusti (Lugo), Marqués de Valdecilla (Santander), Bellvitge (Barcelona), San Cecilio (Granada), Canarias (Tenerife), Doctor Peset (Valencia), General de Ciudad Real (Ciudad Real) and Clínico San Carlos, La Paz, La Princesa, Gregorio Marañón and 12 de Octubre (Madrid). This cohort included patients with RA from all parts of Spain. For this purpose, only native Spaniards were included and individuals with RA of different genetic backgrounds such as South Americans, black people or other individuals from different parts of the world were excluded from the analysis.

For experiments involving humans and the use of human blood samples, all the methods were carried out in accordance with the approved guidelines and regulations, according to the Declaration of Helsinki. All experimental protocols were approved by the Ethics Committees of clinical research of Galicia for Hospital Lucus Augusti in Lugo, of Cantabria for Hospital Marqués de Valdecilla in Santander, of Cataluña for Hospital de Bellvitge in Barcelona, of Andalucía for Hospital San Cecilio in Granada, of Canarias for Hospital de Canarias in Tenerife, of Comunidad Valenciana for Hospital Doctor Peset in Valencia, of Castilla-La Mancha for Hospital General de Ciudad Real in Ciudad Real and of Madrid for Hospital Clínico San Carlos, La Paz, La Princesa, Gregorio Marañón and 12 de Octubre in Madrid. Informed consent was obtained from all subjects.

Epidemiological and clinical characteristics of patients enrolled in the study are shown in Table 1. Definitions of cardiovascular (CV) events and for traditional CV risk factors were established as previously described^8^.

### Genotyping

DNA from patients was obtained from peripheral blood using standard methods.

For the selection of the gene polymorphisms studied in the present report, we carried out a search of genes associated with CVD or subclinical atherosclerosis in the general population. Based on this analysis, *TCF21* rs12190287, *LPA* rs3798220, *HHIPL1* rs2895811, *RASD1-PEMT* rs12936587, *MRPS6* rs9982601, *CYP17A1-CNNM2-NT5C2* rs12413409, *SMG6-SRR* rs216172, *PHACTR1* rs12526453, *WDR12* rs6725887, *COL4A1-COL4A2* rs4773144, *ZHX2* rs11781551, *PINX1* rs6601530, *SLC17A4* rs4712972, *LRIG1* rs17045031 and *LDLR* rs6511720 were assessed in patients with RA. For this purpose, a TaqMan predesigned single-nucleotide polymorphism genotyping assays in a 7900 HT Real-Time polymerase chain reaction (PCR) system, according to the conditions recommended by the manufacturer (Applied Biosystems, Foster City, CA, USA), was performed.

Negative controls and duplicate samples were included to check the accuracy of genotyping.

### Carotid ultrasonography (US) examination

cIMT values and presence/absence of carotid plaques were evaluated in 1,258 cases. Patients from Santander, Granada, Tenerife, Valencia, Ciudad Real and Madrid were assessed using a commercially available scanner, Mylab 70, Esaote (Genoa, Italy)^9^. Patients from Lugo were assessed using high-resolution B-mode ultrasound, Hewlett Packard SONOS 5500^8^. cIMT was measured at the far wall of the right and left common carotid arteries, 10 mm from the carotid bifurcation, over the proximal 15 mm-long segment. cIMT value was determined as the average of three measurements in each common carotid artery. The final cIMT was the largest average cIMT (left or right)^9^,^10^. The plaque criteria in the accessible extracranial carotid tree were focal protrusion in the lumen at least cIMT >1.5 mm, protrusion at least 50% greater than the surrounding cIMT, or arterial lumen encroaching >0.5 mm^10^,^11^. Agreement between these two US methods was previously reported^12^. Experts with a high reproducibility, excellent inter-observer reliability and close collaboration in the assessment of subclinical atherosclerosis in RA performed the studies.

### Statistical analysis

Genotype data were checked for deviation from Hardy-Weinberg equilibrium (HWE) using http://ihg.gsf.de/cgi-bin/hw/hwa1.pl.

Power for the study was calculated using “CaTS-Power Calculator for Two Stage Association Studies” (http://www.sph.umich.edu/csg/abecasis/CaTS/).

The relationship between allelic frequencies and the presence/absence of CV events was tested using logistic regression adjusting for sex, age at RA diagnosis, follow-up time and traditional CV risk factors as potential confounder factors. Results were expressed as odds ratios (OR) with 95% confidence intervals (CI).

Association between allelic frequencies and cIMT values was tested using unpaired t test. Results were adjusted for sex, age at the time of US study, follow-up time and traditional CV risk factors as potential confounder factors using analysis of covariance (ANCOVA).

Differences in the allelic frequencies according to the presence/absence of carotid plaques were calculated by χ2 or Fisher tests. Strength of associations was estimated using OR and 95% CI. Results were adjusted for sex, age at the time of US study, follow-up time and traditional CV risk factors as potential confounder factors by logistic regression.

Analyses were performed with STATA statistical software 12/SE (Stata Corp., College Station, TX, USA).

## Results

Polymorphisms genotyped were in HWE and genotyping success was >99%.

The study had ≥90% of power to detect genotypic OR = 1.3 for *TCF21* rs12190287, *HHIPL1* rs2895811, *RASD1-PEMT* rs12936587, *SMG6-SRR* rs216172, *PHACTR1* rs12526453, *COL4A1-COL4A2* rs4773144, *ZHX2* rs11781551 and *PINX1* rs6601530, and ≥90% to detect OR ≥1.4 for *LPA* rs3798220, *MRPS6* rs9982601, *CYP17A1-CNNM2-NT5C2* rs12413409, *WDR12* rs6725887, *SLC17A4* rs4712972, *LRIG1* rs17045031 and *LDLR* rs6511720.

### Influence of *TCF21, LPA, HHIPL1, RASD1-PEMT, MRPS6, CYP17A1-CNNM2-NT5C2, SMG6-SRR, PHACTR1, WDR12* and *COL4A1-COL4A2* polymorphisms on CV events or subclinical atherosclerosis in patients with RA

Firstly, we assessed the potential influence of *TCF21* rs12190287, *LPA* rs3798220, *HHIPL1* rs2895811, *RASD1-PEMT* rs12936587, *MRPS6* rs9982601, *CYP17A1-CNNM2-NT5C2* rs12413409, *SMG6-SRR* rs216172, *PHACTR1* rs12526453, *WDR12* rs6725887 and *COL4A1-COL4A2* rs4773144 on the risk of CV events or subclinical atherosclerosis in RA patients (Table 2). In this sense, no statistically significant differences were found when each polymorphism was assessed according to the presence/absence of CV events after adjustment of the results for potential confounder factors. Similarly, no statistically significant differences were detected when each polymorphism was evaluated according to the cIMT values and the presence/absence of carotid plaques in RA patients, even after adjustment (Table 2).

### Influence of *ZHX2, PINX1, SLC17A4, LRIG1* and *LDLR* polymorphisms on CV events or subclinical atherosclerosis in patients with RA

Subsequently, we evaluated the potential relationship between *ZHX2* rs11781551, *PINX1* rs6601530, *SLC17A4* rs4712972, *LRIG1* rs17045031 and *LDLR* rs6511720 and CV events or subclinical atherosclerosis in patients with RA (Table 3). In this regard, no significant differences were obtained when RA patients were stratified according to the presence/absence of CV events, after adjustment of the results for potential confounders (Table 3). It was also the case when RA patients were stratified according to the data derived from the evaluation of the cIMT and the presence/absence of carotid plaques, even after adjustment (Table 3).

## Discussion

Results from our study show that a large number of gene polymorphisms associated with CV disease or subclinical atherosclerosis in the general population are not implicated in the increased risk or CV disease found in Caucasian individuals with RA, which is the prototype of chronic inflammatory rheumatic disease. Since a recent study that included a smaller series of North-American patients with RA found no association with CV disease of most of the gene polymorphisms assessed in our study, our results are also confirmatory and they further enhance the fact that the genetic predisposition for CV disease in RA may not be the same as that of the general population.

Similarities between atherosclerosis and chronic inflammatory diseases have been reported^3^. This is especially true for RA^3^,^13^. In this respect, recruitment of blood mononuclear cells, up-regulation of adhesion molecules and production of pro-inflammatory cytokines and matrix-degrading enzymes were described as potential common mechanisms involved in the initiation and perpetuation of both pathologies^3^. However, as pointed out before, a recent study performed in North-American patients with RA failed to demonstrate the implication of a series of pro-atherogenic genes, which were associated with CV disease in the general population, in the development of CVD in RA^5^.

According to our data, and in contrast to the general population^4^, *TCF21, LPA, HHIPL1, RASD1-PEMT, MRPS6, CYP17A1-CNNM2-NT5C2, SMG6-SRR, PHACTR1, WDR12, COL4A1-COL4A2* polymorphisms are not associated with the development of CV events and the risk of subclinical atherosclerosis in patients with RA.

In our study, we also aimed to determine if other gene polymorphisms implicated in the increased risk of subclinical atherosclerosis in non-rheumatic Caucasians individuals^6^ could also influence the risk for CVD in RA. Unfortunately, unlike non-rheumatic Caucasians^6^, we did not disclose a relationship between *ZHX2, PINX1, SLC17A4* 2, *LRIG1* and *LDLR* variants and CVD in patients with RA.

Taken together, our results suggest that RA itself should be considered an independent CV risk factor. Noteworthy, a former report from our group already highlighted genetic differences between the atherosclerosis disease in the general population and that associated to RA^14^. Consequently, our findings further support that the genetic component implicated in the development of atherosclerosis in RA may be different from that associated to “idiopathic” atherosclerosis. In this line, factors that are intrinsic to RA may independently contribute to the development of CVD.

In conclusion, our results do not confirm an association of *TCF21, LPA, HHIPL1, RASD1-PEMT, MRPS6, CYP17A1-CNNM2-NT5C2, SMG6-SRR, PHACTR1, WDR12, COL4A1-COL4A2, ZHX2, PINX1, SLC17A4* 2, *LRIG1* and *LDLR* with CVD in patients with RA.

## Additional Information

**How to cite this article**: López-Mejías, R. *et al*. Influence of coronary artery disease and subclinical atherosclerosis related polymorphisms on the risk of atherosclerosis in rheumatoid arthritis. *Sci. Rep.* **7**, 40303; doi: 10.1038/srep40303 (2017).

**Publisher's note:** Springer Nature remains neutral with regard to jurisdictional claims in published maps and institutional affiliations.

## Figures and Tables

**Table 1:** Epidemiological and clinical characteristics of the 2,609 Spanish patients with rheumatoid arthritis included in the study.

Clinical Feature	% (n/N)
Age at the time of disease onset (years, mean ± standard deviation)	50.8 ± 14.7
Follow-up (years, mean ± standard deviation)	12.7 ± 8.6
Percentage of women	76.4
Rheumatoid factor positive*	64.7 (1,433/2,213)
Anti-CCP antibodies positive	59.2 (1,333/2,251)
Erosions	53.1 (1,162/2,186)
Extra-articular manifestations†	24.7 (444/1,799)
Cardiovascular risk factors	
Hypertension	37.9 (934/2,462)
Diabetes mellitus	12.5 (308/2,462)
Dyslipidemia	37.9 (935/2,462)
Obesity	21.2 (522/2,462)
Smoking habit	37.0 (911/2,462)
Patients with cardiovascular events	15.7 (410/2,609)
Ischemic heart disease	6.8 (179/2,609)
Heart failure	5.7 (149/2,609)
Cerebrovascular accident	4.5 (118/2,609)
Peripheral arteriopathy	1.9 (51/2,609)

Anti-CCP antibodies: Anti-cyclic citrullinated peptide antibodies.

^*^At least two determinations were required.

^†^If patients experienced: nodular disease, Felty’s syndrome, pulmonary fibrosis, rheumatoid vasculitis, or secondary Sjögren’s syndrome.

**Table 2:** Association between *TCF21, LPA, HHIPL1, RASD1–PEMT, MRPS6, CYP17A1-CNNM2-NT5C2, SMG6-SRR, PHACTR1, WDR12* and *COL4A1-COL4A2* polymorphisms and CV events or subclinical atherosclerosis in patients with RA.

	Change	Presence/absence of CV events (n = 2,609)		cIMT (n = 1,258)	Presence/absence of carotid plaques (n = 1,258)	
		P*	OR (95% CI)*	P†	P‡	OR (95% CI)‡
*TCF21* rs12190287	C/G	0.32	0.87 (0.66–1.14)	0.46	0.58	0.94 (0.77–1.15)
*LPA* rs3798220	T/C	0.15	1.90 (0.79–4.58)	0.14	0.52	1.23 (0.65–2.36)
*HHIPL1* rs2895811	T/C	0.79	1.03 (0.79–1.35)	0.23	0.81	0.97 (0.79–1.19)
*RASD1-PEMT* rs12936587	G/A	0.53	1.08 (0.84–1.40)	0.56	0.43	1.08 (0.89–1.31)
*MRPS6* rs9982601	C/T	0.73	1.07 (0.71–1.61)	0.53	0.59	0.92 (0.67–1.25)
*CYP17A1-CNNM2-NT5C2* rs12413409	G/A	0.55	0.87 (0.56–1.36)	0.24	0.67	1.07 (0.78–1.48)
*SMG6-SRR* rs216172	G/C	0.85	0.97 (0.75–1.27)	0.14	0.31	0.90 (0.74–1.10)
*PHACTR1* rs12526453	C/G	0.86	0.97 (0.74–1.27)	0.83	0.19	1.14 (0.93–1.39)
*WDR12* rs6725887	T/C	0.16	1.28 (0.90–1.82)	0.08	0.24	0.85 (0.65–1.11)
*COL4A1-COL4A2* rs4773144	A/G	0.93	1.01 (0.78–1.30)	0.89	0.62	0.95 (0.78–1.15)

CV: cardiovascular; RA: rheumatoid arthritis; cIMT: carotid intima-media thickness; OR: odds ratio; CI: confidence interval.

^*^Adjusted for sex, age at RA diagnosis, follow-up time and traditional CV risk factors using logistic regression.

^†^Adjusted for sex, age at the time of ultrasonography study, follow-up time and traditional CV risk factors using analysis of covariance (ANCOVA).

^‡^Adjusted for sex, age at the time of ultrasonography study, follow-up time and traditional CV risk factors by logistic regression.

**Table 3:** Association between *ZHX2, LRIG1, PINX1, SLC17A4* and *LDLR* polymorphisms and CV events or subclinical atherosclerosis in patients with RA.

	Change	Presence/absence of CV events (n = 2,609)		cIMT (n = 1,258)	Presence/absence of carotid plaques (n = 1,258)	
		P*	OR (95% CI)*	P†	P‡	OR (95% CI)‡
*ZHX2* rs11781551	G/A	0.96	1.00 (0.77–1.31)	0.37	0.24	0.88 (0.72–1.08)
*PINX1* rs6601530	A/G	0.42	0.90 (0.69–1.16)	0.35	0.76	1.03 (0.85–1.25)
*SLC17A4* rs4712972	G/A	0.07	1.41 (0.97–2.00)	0.99	0.98	0.99 (0.75–1.31)
*LRIG1* rs17045031	G/A	0.72	1.12 (0.59–2.12)	0.35	0.42	0.81 (0.49–1.35)
*LDLR* rs6511720	G/T	0.25	0.81 (0.56–1.16)	0.08	0.46	1.10 (0.85–1.44)

CV: cardiovascular; RA: rheumatoid arthritis; cIMT: carotid intima-media thickness; OR: odds ratio; CI: confidence interval.

^*^Adjusted for sex, age at RA diagnosis, follow-up time and traditional CV risk factors using logistic regression.

^†^Adjusted for sex, age at the time of ultrasonography study, follow-up time and traditional CV risk factors using analysis of covariance (ANCOVA).

^‡^Adjusted for sex, age at the time of ultrasonography study, follow-up time and traditional CV risk factors by logistic regression.
